# Supplementary material for: Effects of Rhizobia Isolated from Coffee Fields in the High Jungle Peruvian Region, Tested on Phaseolus vulgaris L. var. Red Kidney
Source: Microorganisms. 2022 Apr 15;10(4):823. doi: 10.3390/microorganisms10040823 (PMC9027962; doi:10.3390/microorganisms10040823)
Supplement: Supplementary file 1 [file microorganisms-10-00823-s001.zip › microorganisms-1625357-supplementary.pdf]

microorganisms

MDPI

Article

Effects of Rhizobia Isolated from Coffee Fields in the Peruvian Region on *Phaseolus vulgaris* L. Var. Red Kidney Bean Yield

Jesus Lirio-Paredes, Katty Ogata-Gutiérrez and Doris Elizabeth Zúñiga-Dávila *

Laboratorio de Ecología Microbiana y Biotecnología, Department of Biology, Faculty of Sciencie, Universidad Nacional Agraria La Molina, Peru; jesusliriopar@gmail.com (J.L.-P.); kogata@lamolina.edu.pe (K.O.-G.)
* Correspondence: dzuniga@lamolina.edu.pe

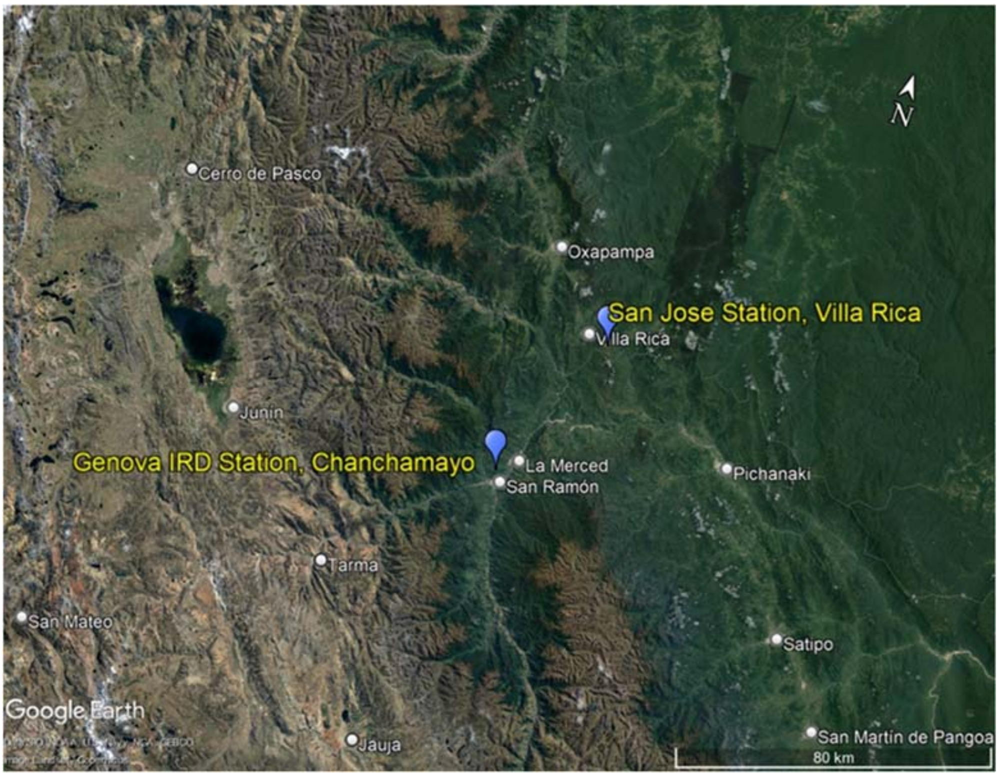

Figure S1. Map to locate the two sampling areas.
